# *O*-GlcNAcylation and Metabolic Reprograming in Cancer

**DOI:** 10.3389/fendo.2014.00145

**Published:** 2014-09-09

**Authors:** Paweł Jóźwiak, Ewa Forma, Magdalena Bryś, Anna Krześlak

**Affiliations:** ^1^Department of Cytobiochemistry, Faculty of Biology and Environmental Protection, University of Lodz, Lodz, Poland

**Keywords:** *O*-GlcNAcylation, cancer, metabolism, PI3K/Akt pathway, transcription factors, glycolytic enzymes, epigenetics

## Abstract

Although cancer metabolism has received considerable attention over the past decade, our knowledge on its specifics is still fragmentary. Altered cellular metabolism is one of the most important hallmarks of cancer. Cancer cells exhibit aberrant glucose metabolism characterized by aerobic glycolysis, a phenomenon known as Warburg effect. Accelerated glucose uptake and glycolysis are main characteristics of cancer cells that allow them for intensive growth and proliferation. Accumulating evidence suggests that *O*-GlcNAc transferase (OGT), an enzyme responsible for modification of proteins with *N*-acetylglucosamine, may act as a nutrient sensor that links hexosamine biosynthesis pathway to oncogenic signaling and regulation of factors involved in glucose and lipid metabolism. Recent studies suggest that metabolic reprograming in cancer is connected to changes at the epigenetic level. *O*-GlcNAcylation seems to play an important role in the regulation of the epigenome in response to cellular metabolic status. Through histone modifications and assembly of gene transcription complexes, OGT can impact on expression of genes important for cellular metabolism. This paper reviews recent findings related to *O*-GlcNAc-dependent regulation of signaling pathways, transcription factors, enzymes, and epigenetic changes involved in metabolic reprograming of cancer.

## Cancer Cell Metabolism

Most early studies concerning cancer biology focused only on molecular alterations in signaling pathways that led to uncontrolled proliferation, while changes in cancer metabolism were treated as a secondary effect. However, in recent years, a growing body of evidence has demonstrated that metabolic reprograming can be a key process during tumorigenesis and many oncogenes and tumor suppressors are, in fact, regulators of metabolism. Changes in metabolism are necessary for the shift from normal to malignant growth (1).

**Figure 1:**
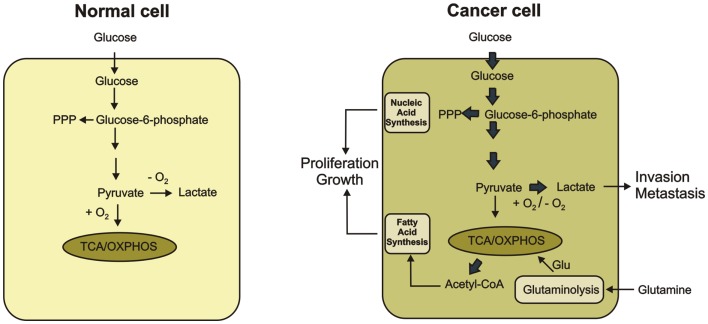
**Metabolic differences between normal and cancer cells are shown**. In normal cells, glucose is metabolized to pyruvate, which is completely oxidized to CO_2_ through the TCA cycle and the oxidative phosphorylation process in the mitochondria. Only if O_2_ is limited, pyruvate is metabolized to lactate. Cancer cells convert most glucose to lactate regardless of the availability of O_2_ (the Warburg effect). Secretion of lactate favors tumor progression. The increased glucose utilization through glycolytic pathway generates metabolic intermediates such as glucose-6-phosphate, which is used for the synthesis of nucleic acids through the pentose phosphate pathway. Glutamate produced during glutaminolysis serves as the major substrate to refuel the TCA cycle. Citrate-derived acetyl CoA is used for lipid production. The increased synthesis of nucleic acid and lipids promote proliferation and growth of cancer cells.

Cancer cell metabolism is characterized by an enhanced uptake and utilization of glucose (2–6). In normal cells, glucose is catabolized to pyruvate. Pyruvate is further converted to acetylo-CoA and oxidized to carbon dioxide through the mitochondrial tricarboxylic acid (TCA) cycle, which generates NADH and FADH_2_. The transfer of electrons from NADH and FADH_2_ to oxygen through respiratory chain is an energy-efficient process. Together, glycolysis, TCA cycle, and electrons transfer phosphorylation produce 36 ATP molecules per glucose molecule. In cancer cells, oxidative phosphorylation is inhibited and cells use glycolysis to provide them with the necessary energy. Glycolysis can only provide 2 ATP molecules per glucose molecule producing lactic acid as the end product. Cancer cells preferentially use glycolysis even in the abundance of oxygen whereas normal cells use only when oxygen supply is limited (4–6). The increased glucose uptake with concomitant lactate production, even under aerobic conditions, is known as the Warburg effect or aerobic effect (2, 3) (Figure 1).

It was originally hypothesized that these metabolic changes in cancer cells reflected damage to mitochondrial oxidative phosphorylation, suggesting that cancer cells are forced to use glycolysis instead of oxidative phosphorylation (1–3). However, it has been revealed that many cancer cells are capable of synthesizing ATP through mitochondrial respiration (7, 8). There is also no strong evidence that respiration is less active in cancer cells than in normal cells. Additionally, mitochondria play important role in cancer because they are involved in biosynthesis of molecules necessary for growth and proliferation. Impairment of mitochondrial function has been shown to suppress tumor growth (9). Therefore, increased glycolysis is not just a consequence of impaired mitochondria but rather constitutes a primary change of cancer metabolism.

In fact, increased glycolytic flux is very beneficial to cancer cells because the glycolytic intermediates fuel several biosynthetic pathways that produce *de novo* nucleotides, lipids, amino acids, and NADPH. Reprograming of cellular metabolism toward synthesis of precursors for macromolecules allows for the accumulation of biomass during cell growth and proliferation (10, 11). Moreover, cancer cells are more resistant to hypoxia condition associated with tumor growth by switching their metabolism from oxidative phosphorylation to oxygen-independent glycolysis (12). By producing an increased amount of lactic acid, cancer cells can lower the pH of extracellular microenvironment, which induces the activity of metalloproteases and facilitates degradation of extracellular matrix components. Thus, lactate can be an inducer of cancer invasion and metastasis (13–15) (Figure 1).

The molecular mechanisms that control metabolic reprograming in cancer cells are complex. Tumors conduct aerobic glycolysis and upregulate glutaminolysis, lipid metabolism, and pentose phosphate pathway (PPP), partly through the activation of oncogenes or loss of tumor suppressor activity. Oncogenes such as Akt or c-Myc are promoters of cancer metabolic changes. In contrast, tumor suppressors such as p53 or AMP-activated protein kinase (AMPK) prevent those alterations (6, 16, 17). It is also suggested that epigenetic changes may contribute to the Warburg effect (18).

## *O*-GlcNAcylation

**Figure 2:**
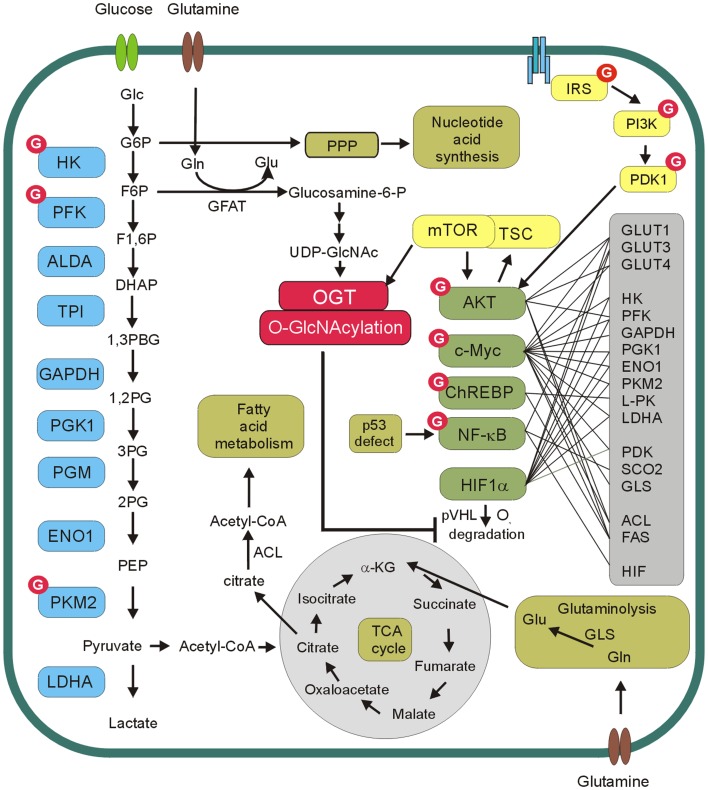
***O*-GlcNAc and cancer metabolism**. *O*-GlcNAcylation level of proteins is dependent on concentration of UDP-*N*-acetylglucosamine (UDP-GlcNAc), which is a donor substrate for *O*-GlcNAc transferase (OGT). UDP-GlcNAc is derived from glucose through hexosamine biosynthetic pathway. In this pathway, fructose-6-phospate (F6P) is converted to glucosamine-6-phosphate by the glutamine:fructose-6-phosphate amidotransferase (GFAT) and after the subset of reactions UDP-GlcNAc is generated. OGT modifies and regulates several glycolytic enzymes, transcription factors as well as components of PI3K/Akt/mTOR pathway. Akt, c-Myc, ChREBP, NF-kB, and HIF-1α reprogram cellular metabolism by direct or indirect regulation of expression of glucose transporters (GLUT1, GLUT3, GLUT4), glycolytic enzymes (HK, PFK, GAPDH, PGK1, ENO1, PKM2, L-PK, LDHA), pyruvate dehydrogenase kinase (PDK), glutaminase (GLS), cytochrome *c* oxidase 2 (SCO2), fatty acid synthase (FAS), ATP citrate lyase (ACL).

*O*-GlcNAcylation is a post-translational modification of cellular proteins that is suggested to play a role in the nutrient sensing mechanism (19, 20). This modification results from the enzymatic addition of the *N*-acetylglucosamine (GlcNAc) moiety to the hydroxyl groups of serines or threonines. *O*-GlcNAcylation is dynamically regulated by *O*-GlcNAc transferase (OGT) and *O*-GlcNAcase (OGA), which are respectively responsible for *O*-GlcNAc addition and removal (21, 22). *O*-GlcNAc modification level of proteins is dependent on the concentration of UDP-GlcNAc, which is a donor substrate for OGT. UDP-GlcNAc is the end product of the hexosamine biosynthetic pathway (HBP), which directly uses cell glucose input. Consequently, *O*-GlcNAcylation is modulated by nutrients availability. Therefore, *O*-GlcNAcylation is proposed as a nutrient sensor and metabolic regulator (19, 20, 23). Glucose and glutamine are the two most abundant extracellular nutrients and cancer cells are highly dependent on availability of these compounds. Glutamine is the donor substrate in the conversion of fructose-6-phosphate to glucosamine-6-phosphate by glutamine:fructose-6-phosphate amidotransferase (GFAT) in the HBP. Thus, an excess in both glutamine and glucose uptake in cancer cells contributes to an increased flux through the HBP. This in turn contributes to an increased level of the HBP end product, i.e., UDP-GlcNAc and increased *O*-GlcNAcylation (19, 20, 23) (Figure 2).

*O*-GlcNAcylation occurs on serine or threonine residues of proteins at sites that may also be phosphorylated. Therefore, extensive crosstalk exists between phosphorylation and *O*-GlcNAcylation. At first, it was suggested that *O*-GlcNAcylation is a reciprocal to phosphorylation and these modifications are mutually exclusive. However, recent studies have shown that some cellular stimuli are able to increase both modifications on the same proteins. Thus, the interplay between *O*-GlcNAcylation and phosphorylation is more complex than previously assumed (24).

The results of many studies suggest that increased expression of OGT and hyper-*O*-GlcNAcylation are the universal features of cancers [for review see Ref. (25–27)]. Aberrant *O-*GlcNAcylation seems to be involved both in tumorigenesis and cancer progression. *O-*GlcNAcylation of oncogenes, tumor suppressors, and other proteins involved in cell signaling pathways may significantly impact tumor growth, cell proliferation, angiogenesis, invasion, and metastasis. A growing body of evidence suggests that hyper-*O-*GlcNAcylation may also be an important factor in reprograming of cancer cell metabolism (Figure 2).

## Impact of *O*-GlcNAcylation on Key Factors in Cancer Metabolism

### PI3K/Akt/mTOR pathway

Phosphatidylinositol 3-kinase/Akt/mTOR signaling pathway is a key mechanism involved in both growth and glucose metabolism control in cells. Constitutively activated PI3K/Akt/mTOR signaling as a consequence of *PIK3CA* mutations or *PTEN* loss is one of the most common lesion in human cancers (28–30).

**Figure 3:**
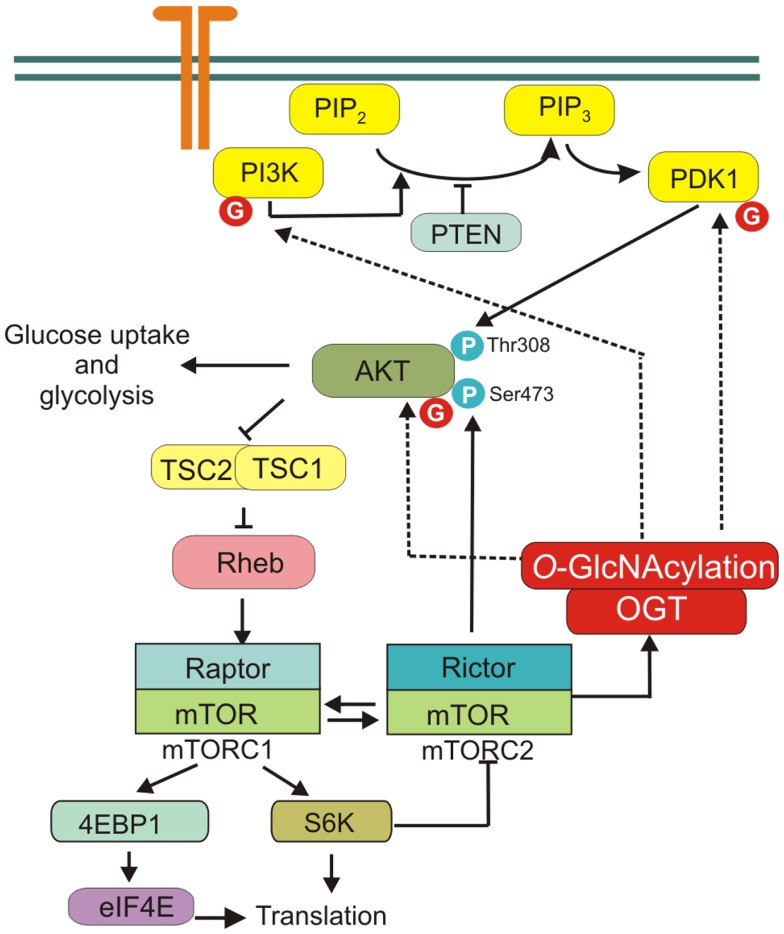
**PI3K/Akt/mTOR signaling pathway**. mTORC1 is activated by receptor signaling through the PI3K/Akt pathway. mTORC2 is crucial factor in PI3K/Akt signaling, phosphorylating Akt on Ser473 to promote its maximal activation. mTOR regulates protein *O*-GlcNAcylation through affecting OGT stability. Several proteins of PI3K/Akt/mTOR pathway are modified by OGT, i.e., PI3K, PDK1, and Akt.

The activation of phosphatidylinositol 3-kinase (PI3K) leads to the phosphorylation of phosphatidylinositol 4,5-bisphosphate to phosphatidylinositol 3,4,5-trisphosphate and subsequent recruitment of Akt to the plasma membrane where this kinase is activated (31). Akt is partially activated through an initial phosphorylation at Thr308 by phosphoinositide-dependent kinase-1 (PDK1) and then fully activated by the phosphorylation at Ser473 by a mammalian target of rapamycin complex 2 (mTORC2) (32–36). Akt can directly or indirectly affect the activity of many transcription factors and enzymes mediating multiple effects (35, 36). One of the major downstream effectors of Akt is the serine/threonine kinase mTOR. mTOR constitutes catalytic subunit of the functionally distinct mTORC1 and mTORC2 complexes. Akt can activate mTORC1 indirectly through phosphorylation and inhibition of tuberous sclerosis complex 2 (TSC2) (37) (Figure 3).

Phosphatidylinositol 3-kinase/Akt/mTOR signaling pathway plays a central role in cancer cell metabolism reprograming (30). PI3K/Akt pathway regulates glucose uptake and utilization (5). Activation of PI3K/Akt causes increased glucose transporters expression on the cell surface, activation of hexokinase (HK) that phosphorylates glucose to keep it in cell and phosphofructokinase-2-dependent allosteric activation of phosphofructokinase-1 (PFK1), which catalyzes the committed step of glycolysis (1). Moreover, activation of PI3K/Akt/mTOR pathway enhances the biosynthesis of macromolecules. PI3K and Akt stimulate expression of lipogenic genes and lipid synthesis in many cell types, while mTOR regulates protein translation (38–40).

The role of *O*-GlcNAcylation in regulation of PI3K/Akt signaling pathway was extensively studied especially in adipocytes and muscle cells (41–45). It was shown that overexpression of OGT and increased *O*-GlcNAcylation in muscle, adipocytes, or liver cells inhibited insulin signaling (23, 43, 46, 47). However, studies using OGA inhibitors gave contradictory results. Inhibition of OGA by PUGNAc [*O*-(2-acetamido-2-deoxy-d-glucopyranosylidene)amino-*N*-phenylcarbamate] increased global *O*-GlcNAc levels and caused insulin resistance in 3T3-L1 adipocytes and skeletal muscle (40, 43). But the other studies showed that more selective than PUGNAc inhibitor NButGT (1,2-dideoxy-2′-propyl-alpha-d-glucopyranoso-[2,1-d]-Delta 2′-thiazoline) did not induce insulin resistance in 3T3-L1 adipocytes (48, 49).

Akt is one of the most frequently investigated *O*-GlcNAcylated proteins. In murine pancreatic β-cells, Akt1 Ser473 may undergo both phosphorylation and *O*-GlcNAcylation and the balance between these modifications may regulate cell apoptosis (50). However, the relationship between *O*-GlcNAcylation and phosphorylation of Akt in cancer cells is not fully elucidated. Wang et al. showed that *O*-GlcNAcylations at Thr305 and Thr312 inhibited Akt phosphorylation at Thr308 via disrupting the interaction between Akt and PDK1 in MCF-7 cells (51). The impaired Akt activation affected functions of Akt, as evidenced by suppressed cell proliferation and migration capabilities. On the other hand, Kanwal et al. showed that in MCF-7 cells treated with PUGNAc and glucosamine the phosphorylation of Akt Ser473 was higher (52). Similarly, in thyroid anaplastic cancer cells, down-regulation of OGA and increased *O*-GlcNAcylation caused increased Akt1 Ser473 phosphorylation and enhanced proliferation (53). Onodera et al. found that OGT inhibition by BADGP (benzyl-2-acetamido-2-deoxy-α-d-galactopyranoside) or down-regulation by siRNA led to suppression of Akt signaling in 3D cultures of breast cancer cells (54).

Additionally, PI3K/Akt pathway is sensitive to extracellular glucose. Jones et al. have shown that short-term glucose deprivation significantly restricts insulin-stimulated Akt activation and inhibits growth of U2OS cancer cells (55). The authors found that insulin signaling can be rescued by extracellular glucosamine and increased flux through the HBP and *O*-GlcNAcylation (55). Together, these data seem to support the concept that in cancer cell metabolism, reprograming increased HBP flux and *O-*GlcNAcylation may play an important role.

Recent studies have also shown that mTOR regulates protein *O*-GlcNAc modification through affecting OGT stability. Inhibition of mTOR causes a decrease in global *O*-GlcNAcylation due to decreased OGT protein level (56).

Thus, many studies have pointed to *O*-GlcNAcylation as a key regulatory modification of PI3K/Akt/mTOR pathway. But further studies are necessary to provide direct evidence for the role of *O*-GlcNAcylation in PI3K/Akt/mTOR pathway in cancer metabolism regulation.

### Hypoxia-induced factor

**Figure 4:**
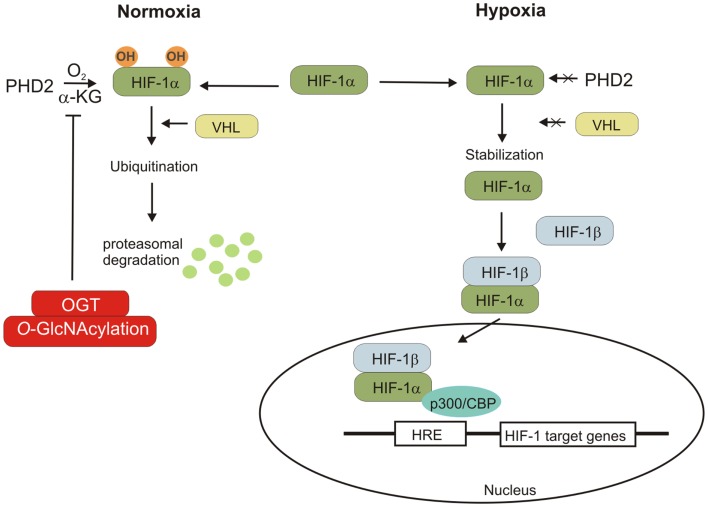
**Hypoxia-inducible factor regulation is shown**. Under normal condition, HIF-1α subunit hydroxylated by PDH2 can bind to VHL protein, which promotes the polyubiquitination of HIF-1α and its degradation. The lack of oxygen prevents the hydroxylation of HIF-1α, leading to its stabilization. HIF-1α can associate with HIF-1β and the cofactor p300/CBP. The HIF-1 complex induces the transcription of genes containing hypoxia-responsive elements (HRE). OGT regulates stability of HIF-1α via regulation of α-ketoglutarate levels and inhibiting HIF-1α hydroxylation.

Hypoxia is an important characteristic of the tumor microenvironment (57–59). Decreased oxygen availability stimulates cells to consume more glucose and produce lactate (59). This adaptive response to reduced O_2_ availability is mediated by hypoxia-induced factors 1 and 2 (HIF-1 and HIF-2). These factors are composed of the constitutively expressed HIF-1β subunit and either the HIF-1α or HIF-2α subunit, which are stable only in hypoxia conditions (17, 58). HIF-1α is ubiquitously expressed whereas HIF-2α expression is restricted to several cell types. Under normoxic conditions, the HIF-1α subunit undergoes hydroxylation on Pro402 and/or Pro564 by prolyl hydroxylase domain protein 2 (PHD2), which uses O_2_ and α-ketoglutarate (α-KG) as substrates (58). Hydroxylated HIF-1α is recognized by von Hippel–Lindau (VHL) tumor suppressor protein, which recruits an E3-ubiquitin ligase that targets HIF-1α for proteasomal degradation. Under hypoxic conditions, the prolyl hydroxylation reactions are inhibited by O_2_ deprivation and HIF-1α accumulates and dimerizes with constitutively expressed HIF-1β. HIF-1 dimer binds to the hypoxia response element of target genes and causes their transcriptional activation. HIF-1’s targets include *SLC2A1* and *SLC2A3* genes encoding for glucose transporters (GLUT1 and 3, respectively) as well as genes encoding for most of glycolytic enzymes (58) (Figure 4).

Recent studies revealed that *O*-GlcNAcylation may affect cancer metabolism reprograming by regulation of HIF-1 pathway (60). In human breast cancer cells, high level of HIF-α is associated with elevated OGT level. Ferrer et al. showed that reduction of *O*-GlcNAcylation in cells increased HIF-1α hydroxylation and interaction with VHL resulting in HIF-1α degradation and reduction of GLUT1 expression (60).

### c-Myc

c-Myc is a helix–loop–helix leucine zipper transcription factor, which participates in many cellular processes including cell proliferation, apoptosis, and differentiation (61, 62). This transcription factor is also a key regulator of cancer cell metabolism. In transformed cells, c-Myc is often expressed at constitutively high levels and promotes energy production and biomolecule synthesis independent of growth factor stimulation (16). Activated c-Myc induces the expression of almost all glycolytic enzymes, particularly hexokinase 2 (HK2), phosphofructokinase-1 (PFK1), phosphoglycerate kinase-1 (PGK1), lactate dehydrogenase A (LDHA), and pyruvate kinase M2 (PKM2) (17, 63). c-Myc not only promotes energy production by enhancing glycolysis but also increases biomolecule synthesis by targeting genes of anabolic enzymes such as carbamoyl phosphate synthetase aspartate transcarbamylase and dihydroorotase (CAD), serine hydroxymethyl transferase (SHMT), fatty acid synthase (FAS), and ornithine decarboxylase (ODC) (17). Moreover, multiple studies have demonstrated that c-Myc stimulates glutamine uptake and metabolism. c-Myc directly stimulates expression of glutamine transporters and indirectly promotes glutaminase (GLS) activity by repressing expression of miR-23a/b, which targets *GLS1* transcript (64–66). High level of c-Myc in cancer cells causes glutamine addiction, and cells undergo apoptosis when deprived of glutamine (64).

**Figure 5:**
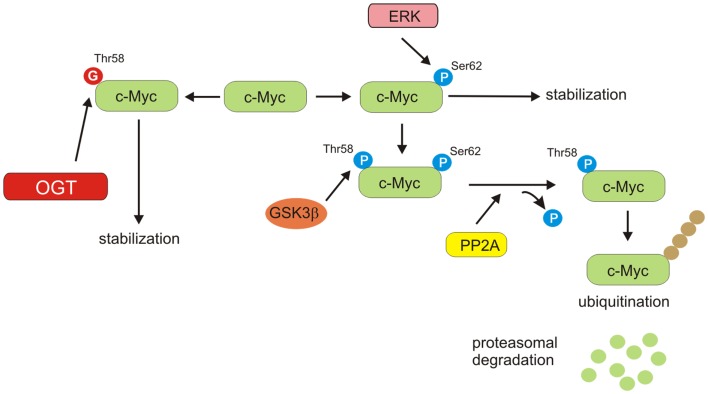
**c-Myc stability regulation**. ERK phosphorylates c-Myc on Ser62, resulting in stabilization of protein. Phosphorylated on Ser62 c-Myc can be modified by GSK3β on Ser58. PP2A can dephosphorylate Ser62. Thr58 mono-phosphorylated c-Myc is a target for ubiquitin ligase complex, leading to proteasomal degradation. *O*-GlcNAcylation of Thr58 can compete with phosphorylation and potentially increase stability of c-Myc.

Stability of c-Myc is controlled by phosphorylation of specific sites (67, 68). Activated extracellular receptor kinase (ERK) stabilizes c-Myc by phosphorylation at Ser62. Once c-Myc phosphorylated at Ser62, it is recognized by GSK3β, which phosphorylates it at Thr58. At that time, dephosphorylation of Ser62 is mediated by protein phosphatase 2A (PP2A) (69). c-Myc phosphorylated at Thr58, but not at Ser62 is recognized by the E3 ligase, which ubiquitinates c-Myc at the N-terminus and targets it for proteasome-dependent degradation (69, 70). Thus, phosphorylation of Thr58 is a key event in c-Myc regulation (Figure 5). Mutation of Thr58 has been observed in Burkitt’s lymphomas and is associated with increased c-Myc protein stability. It was shown that c-Myc could be also *O*-GlcNAcylated at Thr58 (71–73). Increased Thr58 *O*-GlcNAcylation could compete with phosphorylation and potentially stabilize c-Myc. Moreover, PP2A has been found to be *O*-GlcNAcylated in oocytes of *Xenopus laevis* (74). However, the significance of its *O*-GlcNAcylation in cancer cells has not been established. Recently, Itkonen et al. have shown that OGT is, in fact, a central regulator of c-Myc stability in prostate cancer cells (75). OGT inhibition elicited a dose-dependent decrease in the levels of c-Myc protein but not c-Myc mRNA in prostate cell lines (75). Collectively, these data suggest that OGT by modification of c-Myc and PP2A could potentially regulate c-Myc stability and affect its function in cancer cell metabolism.

### NF-κB

**Figure 6:**
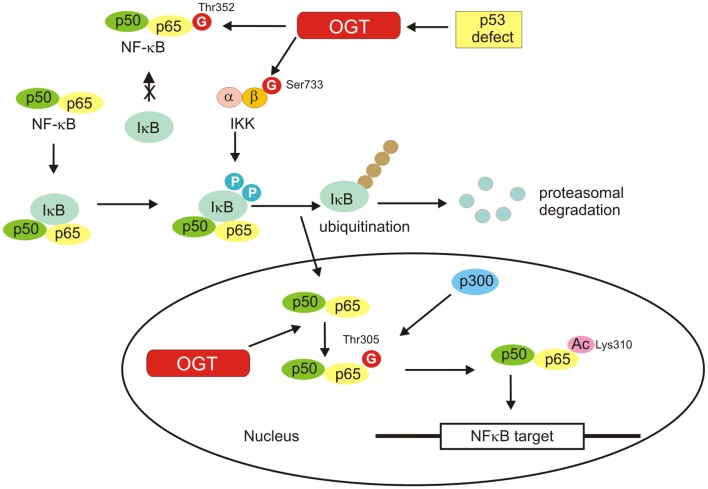
**NF-κB activation is shown**. Inactive NF-κB is located in the cytosol complexed with the inhibitory protein IκB. Activated by extracellular signals IκB kinase (IKK) phosphorylates the IκB protein, which results in dissociation of IκB from NF-κB, ubiquitination, and degradation of IκB by the proteosome. The activated NF-κB is then translocated into the nucleus where it binds to specific sequences of DNA. *O*-GlcNAcylation favors activation of NF-κB by regulation of IKKβ activity, inhibition of the interaction of NF-κB with IkB, and promotion of NF-κB acetylation.

NF-κB is a glucose-responsive transcription factor that is involved in many biological processes such as inflammation and immune response, cell survival, growth, and development (76). Five members of NF-κB transcription factors family have been identified: p65 (RelA), RelB, c-Rel, p105/p50, and p100/p52. Activation of NF-κB proteins is tightly regulated and altered activation of the NF-κB signaling pathways has been linked to autoimmunity, chronic inflammation, and various cancers. In basal state, NF-κB is sequestered by inhibitor of κB (IκB) in the cytosol. Upon stimulation, IκB is phosphorylated by the IκB kinase (IKK) complex and is then degraded by the ubiquitin–proteasome system. The freed NF-κB translocates into the nucleus and induces gene transcription (76, 77) (Figure 6).

It has been suggested that NF-κB may be an important factor promoting the switch of cellular glucose metabolism from oxidative phosphorylation to oxygen-independent glycolysis in tumor cells (18). Kawauchi et al. showed the link between p53, NF-κB, and glycolysis (78). In p53-deficient cells, the activity of NF-κB was found to be enhanced and that caused an increase in the rate of aerobic glycolysis via upregulation of glucose transporter GLUT3 (78). On the other hand, it was found that NF-κB as a regulator of mitochondrial respiration, suppressed reprograming to aerobic glycolysis and prevented necrosis in cells upon nutrient starvation. But this function of NF-κB was p53-dependent and involved upregulation of mitochondrial synthesis of cytochrome *c* oxidase 2, which increased oxidative phosphorylation and reduced glycolytic flux in cells (79, 80). NF-κB is also involved in metabolism via p53-independent mechanisms. Kumar et al. have found that transglutaminase-2 regulates metabolic reprograming in mammary epithelial cells by constitutively activating nuclear factor NF-κB, which binds to hypoxia-inducible factor promoter and induces its transcription even under normoxic conditions (81).

Activation of NF-κB requires post-translational modifications such as phosphorylation and acetylation. Growing evidence also suggests a pivotal role for *O*-GlcNAcylation in the activation of NF-κB (82–86) (Figure 6). The *O*-GlcNAc modification sites within NF-κB p65 have been identified as Thr322 and Thr352. *O*-GlcNAc modification of NF-κB p65 at Thr352 in response to high glucose has been shown to inhibit the interaction of NF-κB with IκB, causing the nuclear translocation of NF-κB and activation of its target genes (83). Recently, Allison et al. have demonstrated that OGT localizes to chromatin and drives p300-mediated acetylation of p65 at Lys310 in response to tumor necrosis factor (TNF) (84). The studies revealed that Thr305 was an important residue required for an attachment of the *O*-GlcNAc moiety on p65. The attachment of the *O-*GlcNAc moiety to p65 at Thr305 is a precondition for Lys310 acetylation, which is necessary for full NF-κB-dependent transcription (84).

IκB kinase is also *O*-GlcNAcylated. Kawauchi et al. showed that loss of p53 enhanced catalytic activity of IKKβ through *O*-GlcNAcylation in mouse embryonic fibroblasts (MEFs) and transformed human fibroblasts (87). *O*-GlcNAcylation of IKKβ occurred at Ser733 in the C-terminal domain, which was identified as an inactivating phosphorylation site. Thus, *O*-GlcNAcylation of IKKβ regulates its catalytic activity (87) (Figure 6).

The direct link between HBP, OGT, and NF-κB was shown in human pancreatic ductal adenocarcinoma cells (PDAC) (85). Ma et al. have observed increased HBP flux and hyper-*O-*GlcNAcylation in PDAC cells, which was associated with increased OGT and decreased OGA levels (85). In these cells, the NF-κB p65 subunit and upstream kinases IKKα/IKKβ were *O*-GlcNAcylated. Reducing p65 *O*-GlcNAcylation specifically by mutating two p65 *O*-GlcNAc sites caused the reduction of PDAC cells anchorage-independent growth (85).

p65 is not the only *O*-GlcNAcylated NF-κB family member. Ramakrishnan et al. examined the *O*-GlcNAcylation status of all of the NF-κβ family proteins in lymphocytes under hyperglycemic conditions (86). They have shown that c-Rel is the major *O*-GlcNAcylated NF-κβ subunit in lymphocytes, and that enhancement of its *O*-GlcNAcylation increases its transcriptional activity. They have identified Ser350 as the site of *O*-GlcNAcylation. Mutation of Ser350 blocked the *O*-GlcNAcylation of c-Rel and greatly reduced DNA-binding ability and transactivation potential in cells in response to stimulation of the T cell receptor (86).

### Carbohydrate responsive element-binding protein

Carbohydrate responsive element-binding protein (ChREBP) is helix–loop–helix leucine zipper transcription factor, which mediates glucose-dependent induction of glycolytic and lipogenic enzyme genes (88–94). ChREBP is involved in the induction of liver pyruvate kinase (L-PK) and acting synergistically with sterol regulatory element-binding protein 1c (SERBP-1c) activates genes encoding lipogenic enzymes: acetyl-CoA carboxylase (ACC) and FAS (88–94). ChREBP is expressed in most tissues but the highest level of this protein is observed in liver and adipocytes (94). The function of ChREBP in hepatocytes has been extensively studied but its role in cancer cell metabolism has not been fully elucidated. However, the studies of Tong et al. suggest that ChREBP plays a key role in regulation of proliferating cells metabolism (95). This study demonstrated that induction of ChREBP in response to mitogenic stimulation was required for proliferation of HCT116 colorectal cancer cells and HepG2 hepatoblastoma cells. Suppression of ChREBP causes a reduction of aerobic glycolysis, *de novo* lipogenesis, and nucleotide biosynthesis but stimulated mitochondrial respiration (95). Thus, ChREBP seems to contribute to the glycolytic phenotype exhibited by cancer cells. It plays a key role in directing glucose metabolism into anabolic pathways, i.e., lipid and nucleotide biosynthesis during cell growth (95).

Carbohydrate responsive element-binding protein contains several phosphorylation sites recognized by protein kinase A (PKA) such as Ser196, Ser626, and Thr666 that are involved in negative regulation of its nuclear import and DNA-binding activity (94). However, mutations of Ser196, Ser626, and Thr666 did not significantly affect the glucose-responsiveness of ChREBP. It appears that PKA-mediated phosphorylation and glucose activation are independent regulatory mechanisms (94).

**Figure 7:**
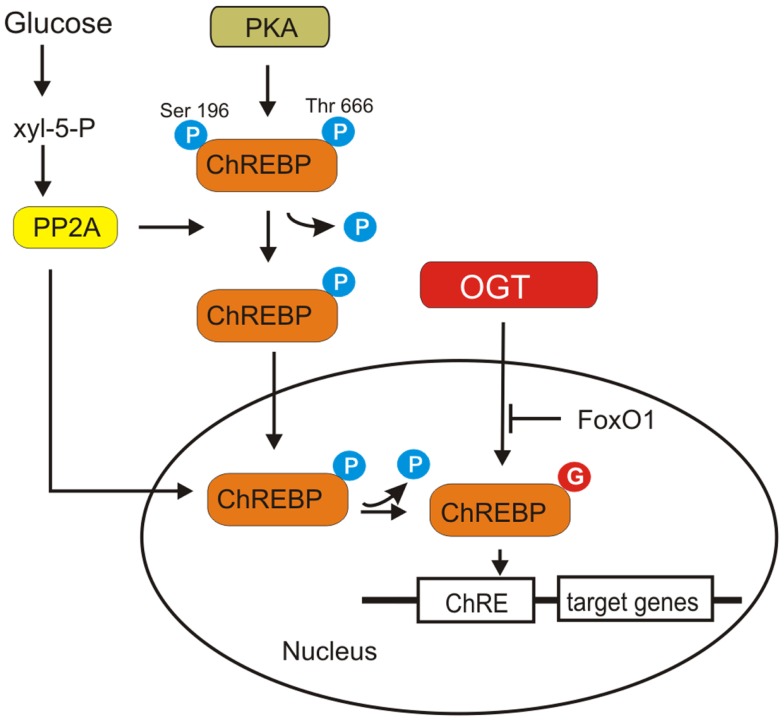
**ChREBP activation is shown**. ChREBP is phosphorylated by PKA. Dephosphorylation of Ser196 and Thr666 ChREBP is required for its translocation into the nucleus and DNA binding. A particular isoform of protein phosphatase 2A (PP2A), selectively activated by Xyl-5-P, an intermediate of pentose phosphate pathway, is responsible for both cytosolic and nuclear dephosphorylation of ChREBP. *O*-GlcNAcylation of ChREBP increases its protein level and transcriptional activity. Overexpression of FoxO1 attenuates ChREBP activity.

Carbohydrate responsive element-binding protein is modified by *O*-GlcNAcylation and this modification increases its protein level and transcriptional activity (96, 97) (Figure 7). *O*-GlcNAcylation affects ChREBP protein stability and protects it against proteasomal degradation. *O*-GlcNAcylated ChREBP under hyperglycemic conditions shows increased activity toward its target glycolytic (*LPK*) and lipogenic (*ACC*, *FAS*, *SCD1*) genes (97).

Ido-Kitamura et al. have shown that FoxO1 is a negative regulator of ChREBP activity (98) (Figure 7). FoxO1 decreases glucose utilization and lipid synthesis by reducing ChREBP activity. Overexpression of FoxO1 in hepatocytes attenuated ChREBP activity by suppressing *O*-GlcNAcylation and reducing the protein stability. FoxO1 inhibits high glucose- or OGT-induced L-PK promoter activity by decreasing ChREBP recruitment to the L-PK promoter (98). However, the exact mechanism by which FoxO1 inhibits ChREBP *O*-GlcNAcylation is not known.

### Glycolytic enzymes

Several glycolytic enzymes related to Warburg effect are *O*-GlcNAcylated (99–101). One of the most important enzymes involved in cancer cell metabolism reprograming is pyruvate kinase (PK) (17). This enzyme catalyzes a reaction generating pyruvate and ATP from phosphoenolpyruvate (PEP) and ADP (102). There are four isozymes of PK (L, R, M1, and M2) and these vary in tissue distribution, kinetic characteristics, and regulatory mechanism. PKL and PKR are products of PKL gene, transcribed with different promoters (103). PKM1 and PKM2 are encoded by the PKM gene and are the products of two alternatively spliced exons (exon 9 and exon 10, respectively) (104). Isozyme M1 is expressed in most adult differentiated tissues, whereas M2 is expressed in embryonic cells, adult stem cells, and cancer cells (105). PKM2 possesses unique properties important in the reprograming of cell metabolism. Active PKs consist of four subunits, and PKL, PKR, and PKM1 form stable tetramers. PKM2 can exist as an active tetramers and much less active dimers (102). When PKM2 is in dimeric form, glycolytic intermediates above PK accumulate and may be directed toward anabolic pathways for synthesis of amino acids, nucleic acids, and phospholipids (17).

*O*-GlcNAcylation may be involved in regulation of PKM2 activity. The site of *O*-GlcNAcylation on PKM2 has not been established. However, the increased *O*-GlcNAcylation in cells is associated with a decrease in general PK activity. It is suggested that hyper-*O*-GlcNAcylation in cancer cells would likely decrease PKM2 activity contributing to directing glycolytic intermediates toward biosynthetic pathways (25). Interestingly, Champattanachai et al. showed that PKM2 is *O*-GlcNAc modified only in breast cancer tissues but not in normal samples (101).

To form the active tetramer, PKM2 requires fructose-1,6-bisphosphate, which is produced in reaction catalyzed by phosphofructokinase-1 (17). Yi et al. have demonstrated that PFK1 is *O*-GlcNAcylated at Ser529 in response to hypoxia in cancer cells (100). Glycosylation inhibits PFK1 activity and redirect the flux of glucose from glycolysis through the PPP (100). Yi et al. have also examined the impact of OGT overexpression on HK, PGK, and PK activities (100). Direct *O*-GlcNAcylation status of these proteins has not been studied but in cancer cells with increased OGT activity, HK activity was increased while PGK and PK activities were decreased (100).

## Epigenetics, *O*-GlcNAcylation, and Cancer Metabolism

The connection between cancer metabolism reprograming and epigenetics may be considered in two aspects. Changes in cancer cell metabolism may impact epigenetic gene regulation since the enzymes involved in modification of histones or chromatin remodeling utilize substrates generated by metabolic pathways (106, 107). On the other hand, through modification and remodeling of chromatin, extracellular signals from tumor microenvironment or nutrition compounds can regulate the expression of genes involved in cellular proliferation as well as cellular metabolism (18).

The studies of Gao et al. have revealed that high glucose is an inducer of monoubiquitination of histone H2B at Lys120 in cultured glioma cells (108). Nutrient deprivation causes decrease of H2B ubiquitination (109). Compared to the other histone modifications, ubiquitination is less well studied and its specific roles in tumors remain to be clarified. However, de-regulation of H2Bub has been suggested as an etiology factor of cancer development (110, 111). The enzymes responsible for H2B monoubiquitination were first identified in *Saccharomyces cerevisiae* as Rad6 (E2) and Bre1 (E3). In humans, there are two homologs of Rad6 (HR6A and HR6B) and Bre1 (RNF20 and RNF40) (110). The latest seem to play main role in ubiquitination of H2B in humans (112, 113). RNF20 physically interacts with the tumor suppressor protein p53, functioning as a transcriptional co-activator of p53 (112). RNF20 is also required for p53 expression and RNF20 depletion leads to more than 10-fold decrease in expression of p53 (114). Monoubiquitination of histone H2B can be reversed by Ubp8, a component of the transcriptional activator SAGA in yeast. USP22 is the human homolog of this protein (110). The results showed also that USP22 is a positive regulator of c-Myc-dependent transcription and induction of c-Myc targeted genes is impaired in USP22-depleted cells (115). Although most data indicate that H2Bub and its ubiquitin ligases act as tumor suppressors, a few studies suggest that their activity may promote tumorigenesis (116–118). The discrepancies found may be due to different role of H2B ubiquitination in tumorigenesis and tumor progression. H2B ubiqiutination may be involved in arising of tumors and proliferation of cancer cells but may suppress cancer stem cell phenotypes. In fact, it has been found that RNF20 and RNF40 knockdown decrease cell proliferation but increase cell migration (111).

Recent studies have also shown that *O*-GlcNAcylation plays an important role in H2B ubiquitination. H2B is *O*-GlcNAcylated by OGT at Ser112 (119–122). H2B Ser112 *O-*GlcNAcylation changes in response to extracellular glucose (120). It is suggested that H2B Ser112 *O-*GlcNAcylation promotes Lys120 monoubiquitination because GlcNAc moiety can serve as an anchor for a histone H2B ubiquitin ligase (122). *O*-GlcNAcylation of H2B is probably important for transcriptional activation since modified by *O-*GlcNAc H2B is frequently located near transcribed genes (120). H2B Ser112 *O*-GlcNAcylation depends on TET2/3 (ten-eleven translocation), which is an enzyme that catalyzes the conversion of 5-methylcytosine to 5-hydoxymethylcytosine (121). TET2 and 3 directly interact with OGT (121, 122). TET2 promotes OGT activity and facilitates OGT-dependent histone modification (121). Xu et al. have found that AMPK could suppress histone H2B *O*-GlcNAcylation (123). AMPK directly phosphorylates OGT and this modification inhibits OGT–chromatin association, histones *O*-GlcNAcylation, and gene transcription. The authors have suggested that there is a crosstalk between the LKB1-AMPK and the hexosamine biosynthesis (HBP)-OGT pathways, which coordinate together for the sensing of nutrient state and regulation of gene transcription (123).

Additionally, it has been recently found that methyltransferase EZH2, which is a component of Polycomb repressive complex 2, is *O*-GlcNAcylated at Ser75 in breast cancer cells (124). This modification stabilizes EZH2 and facilitates the trimethylation of histone H3 at Lys27. Thus, the study of Chu et al. uncovered a unique epigenetic role of OGT in regulating histone methylation (124). It is also possible that OGT by regulation of EZH2 may be involved in metabolic reprograming. Polycomb group protein EZH2 is a direct upstream regulator of c-Myc oncogene (125). c-Myc is one of the main regulators of cancer cell reprograming process. EZH2 was found to activate c-Myc in breast cancer cells through the ERα and the Wnt pathways (126).

*O*-GlcNAcylation plays an important role in activation of NF-κB and this factor seems to be also involved in epigenetic regulation of Warburg effect. Liu et al. have shown that fructose-1,6-bisphosphatase-1 (FBP1), which is gluconeogenesis regulatory enzyme and functions to antagonize glycolysis has been down-regulated through NF-κB pathway in Ras-transformed NIH3T3 cells (127). The authors have found that inhibition of NF-κB restored FBP1 expression, partially through demethylation of FBP1 promoter. NF-kB can be involved in negative regulation of gene expression through interaction with transcription co-repressors such as histone deacetylase HDAC1 and HDAC2 (128, 129). Interestingly, HDAC1 has been found to be *O-*GlcNAcylated in HepG2 liver carcinoma cells (130). It is suggested that OGT can contribute along with HDAC to the repression of genes. Moreover, histone deacetylases can interact with DNA methyltransferases that by methylation of promoters can cause stable silencing of gene expression (18).

## Conclusion

There is no doubt that metabolic reprograming is one of the main hallmarks of cancer cells. The most important changes in cancer metabolism include elevation of glucose uptake and glycolysis, enhanced glutaminolysis, induction of PPP, and upregulation of macromolecule synthesis. These changes are beneficial for cancer proliferation, growth, metastasis, and angiogenesis. Many studies have shown that *O*-GlcNAcylation, which acts as a nutrient sensor, is elevated in different cancers and seems to be responsible for coupling cell metabolic status to signal transduction and transcription. It is strongly suggested that increased glucose flux through HBP and elevated UDP-GlcNAc is a general feature of cancer cells that contributes to hyper-*O*-GlcNAcylation. High activity of OGT as a result of both high substrate level and gene overexpression favors modification of several key factors involved in cancer metabolism reprograming. *O*-GlcNAcylation impacts their stability, activity, localization, interaction with other proteins, and in consequence, enhances their effect on reprograming of cell metabolism. Akt, c-Myc, ChREBP, NF-κB, and HIF-1 affect metabolism by direct or indirect regulation of expression of glucose transporters as well as glutaminolytic, glycolytic, and lipogenic enzymes. OGT and *O*-GlcNAcylation may also constitute a link between nutrient status and epigenetic regulation of gene expression. In response to nutrient availability, OGT may directly affect histone code by attachment of *N*-acetylglucosamine residues. Moreover, OGT can indirectly influence gene expression by interactions with histone modifying enzymes and modulation of their stability and activity. However, although a large body of evidence has demonstrated the significance of *O*-GlcNAcylation in metabolism regulation, there is still much to learn about its role in cancer metabolism reprograming. Elucidating the relationship between O-GlcNAc cycling controlling mechanism and cellular metabolic activity of cancer cells is an exciting challenge for future research.

## Conflict of Interest Statement

The authors declare that the research was conducted in the absence of any commercial or financial relationships that could be construed as a potential conflict of interest.

## Abbreviations

4EBP1, factor 4E binding protein 1; α-KG, alpha ketoglutarate; ACC, acetyl-CoA carboxylase; ACL, ATP citrate lyase; Akt, v-akt murine thymoma viral oncogene; ALDA, aldolase A; AMPK, 5′ adenosine monophosphate-activated protein kinase; BADGP, benzyl-2-acetamido-2-deoxy-α-d-galactopyranoside; CAD, carbamoyl phosphate synthetase aspartate transcarbamylase and dihydroorotase; CBP, CREB binding protein; ChRE, carbohydrate response element; ChREBP, carbohydrate responsive element-binding protein; eIF4E, eukaryotic translation initiation factor 4E; ENO1, enolase; ERK, extracellular signal-regulated kinase; ERα, estrogen receptor α; EZH2, enhancer of zeste homolog 2; F6P, fructose-6-phospate; FADH_2_, reduced flavin adenine dinucleotide; FAS, fatty acid synthase; FBP1, fructose-1,6-bisphosphatase-1; FoxO1, forkhead box protein O1; GAPDH, glyceraldehyde 3-phosphate dehydrogenase; GFAT, glutamine fructose-6-phosphate amidotransferase; Glc, glucose; GlcNAc, *N*-acetylglucosamine; GLS, glutaminase; GLUT, glucose transporter; GSK3β, glycogen synthase kinase 3b; H2Bub, monoubiquitinated H2B; HBP, hexosamine biosynthetic pathway; HDAC1/2, histone deacetylases 1/2; HIF-1, hypoxia-induced factor 1; HK, hexokinase; HR6A/B, human orthologs of the *S.cerevisiae* ubiquitin conjugated enzyme Rad6; IκB, inhibitor of kappa B; IKK, I kappa B kinase; LDHA, lactate dehydrogenase A; LKB1, liver kinase B1; L-PK, liver pyruvate kinase; mTOR, mammalian target of rapamycin; mTORC1/2, mammalian target of rapamycin complex 1/2; NADH, nicotinamide adenine dinucleotide; NButGT, 1,2-dideoxy-2′-propyl-alpha-d-glucopyranoso-[2,1-d]-delta 2′-thiazoline; NF-κB, nuclear factor kappa-light-chain-enhancer of activated B cells; ODC, ornithine decarboxylase; OGA, *O*-GlcNAcase, *O*-GlcNAc hydrolase; OGT, *O*-linked *N*-acetylglucosamine (*O*-GlcNAc) transferase; OXPHOS, oxidative phosphorylation; PDK, pyruvate dehydrogenase kinase; PDK1, phosphoinositide-dependent kinase-1; PEP, phosphoenolpyruvate; PFK, phosphofructokinase; PGK, phosphoglycerate kinase-1; PGM, phosphoglycerate mutase; PHD2, prolyl hydroxylase domain protein 2; PI3K, phosphatidylinositol 3-kinase; PIP_2_, phosphatidylinositol 4,5-bisphosphate; PIP_3,_ phosphatidylinositol 3,4,5-trisphosphate; *PIK3CA*, gene encoding the p110alpha catalytic subunit of PI3K; PKA, protein kinase A; PKM2, pyruvate kinase M2; PP2A, protein phosphatase 2A; PPP, pentose phosphate pathway; PTEN, phosphatase and tensin homolog; PUGNAc, O-(2-acetamido-2-deoxy-d-glucopyranosylidene)amino-*N*-phenylcarbamate; Rheb, Ras homolog enriched in brain; RNF20, ring finger protein 20; S6K, ribosomal protein S6 kinase; SAGA, Spt-Ada-Gcn5 histone acetyltransferase complex; SCD1, stearoyl-CoA desaturase; SHMT, serine hydroxymethyl transferase; *SLC2A1*, solute carrier family 2 member 1, gene encoding GLUT1; *SLC2A3*, solute carrier family 2 member 3, gene encoding GLUT3; SOC2, cytochrome *c* oxidase 2; TCA, tricarboxylic acid cycle; TET, ten–eleven translocation methylcytosine dioxygenase; TNF, tumor necrosis factor; TPI, triose phosphate isomerase; TSC1/2, tuberous sclerosis complex 1/2; Ubp8, ubiquitin-specific peptidase 8; UDP-GlcNAc, Uridine diphosphate *N*-acetylglucosamine; USP22, ubiquitin-specific protease 22; VHL, von Hippel–Lindau tumor suppressor protein; Xyl-5-P, xylulose-5-phosphate.
